# Image Quality Assessment Based on Inter-Patch and Intra-Patch Similarity

**DOI:** 10.1371/journal.pone.0116312

**Published:** 2015-03-20

**Authors:** Fei Zhou, Zongqing Lu, Can Wang, Wen Sun, Shu-Tao Xia, Qingmin Liao

**Affiliations:** 1 Department of Electronic Engineering, Tsinghua University, Beijing, 10084, China; 2 The Shenzhen Key Laboratory of Information Science & Technology/Graduate School at Shenzhen, Tsinghua University, Shenzhen, 518055, China; 3 Digital Productivity Flagship, Commonwealth Scientific and Industrial Research Organisation (CSIRO), Hobart, Australia; University of Chinese Academy of Sciences, CHINA

## Abstract

In this paper, we propose a full-reference (FR) image quality assessment (IQA) scheme, which evaluates image fidelity from two aspects: the inter-patch similarity and the intra-patch similarity. The scheme is performed in a patch-wise fashion so that a quality map can be obtained. On one hand, we investigate the disparity between one image patch and its adjacent ones. This disparity is visually described by an inter-patch feature, where the hybrid effect of luminance masking and contrast masking is taken into account. The inter-patch similarity is further measured by modifying the normalized correlation coefficient (NCC). On the other hand, we also attach importance to the impact of image contents within one patch on the IQA problem. For the intra-patch feature, we consider image curvature as an important complement of image gradient. According to local image contents, the intra-patch similarity is measured by adaptively comparing image curvature and gradient. Besides, a nonlinear integration of the inter-patch and intra-patch similarity is presented to obtain an overall score of image quality. The experiments conducted on six publicly available image databases show that our scheme achieves better performance in comparison with several state-of-the-art schemes.

## Introduction

IQA is of great importance in numerous image-based applications and systems, such as image acquisition, transmission, processing, and display. In some image-based systems, IQA schemes can not only be employed to evaluate the system performance but also be embedded in the system optimization, e.g., image restoration in [1]. Although subjective evaluation is the most reliable way of IQA, it is laborious, expensive, and non-embeddable. Therefore, many researchers have paid tremendous attention to objective IQA, whose goal is to design computational models to predict perceptual visual quality automatically and accurately [2–3]. In this paper, we focus on the problem of FR IQA schemes, where a source image is available. This problem is also known as the evaluation of image fidelity or perceived similarity.

The most conventional FR IQA schemes are mean squared error (MSE) and its variations, involving signal-to-noise ratio (SNR) and peak SNR (PSNR). These metrics are popular due to their simple mathematical expressions. Nevertheless, they correlate poorly with perceived quality rating [4], particularly when distortions are content-dependent [5]. Their main problem lies in ignoring features of the human visual system (HVS).

Since the HVS is the ultimate receiver of the majority of visual signal, it seems hopeful to design computational models and systems to simulate functional components in the HVS [6]. The IQA schemes developed based on this point are known as bottom-top or psychophysics-inspired schemes. However, a complete understanding of the HVS, especially the higher level processes therein, has not been provided nowadays due to the complexity [7], although front-end knowledge about the HVS has been extensively studied. Therefore, some researchers prefer to treat the HVS as a black box and build systems with similar input-output relation as the HVS in the sense of IQA. The IQA schemes inspired by this viewpoint are called top-bottom or overarching-based schemes. Of course, the boundary of the above classification is not sharp [2]. The top-bottom models may take psychophysical features of the HVS into account, and vice versa.

Vision researches discover that several octave spacing radial frequency channels exist in the visual pathway [8]. Accordingly, in many IQA schemes [9–13], both the reference and distorted images are decomposed into multiple channels. Fourier decomposition is used in [9] to measure the visual fidelity of encoded images. In [10], discrete cosine transform is employed to provide the maximum visual quality for the minimum bitrate in the design of quantization matrix. The visual SNR (VSNR) metric [11] estimates the distortion of contrast on each wavelet sub-band. In [12], discrete wavelet transform is also adopted before measuring detail loss and additive impairment. In the scheme named as most apparent distortion (MAD) [13], either Fourier transformation or log-Gabor filtering is performed to decompose images according to distortion levels. Some high-level mechanisms of the HVS can also be included in these schemes, e.g., the use of visual property of global precedence in [11] and [13]. A main benefit of channel decomposition is that it is convenient to incorporate the contrast sensitivity function (CSF), which relates the visual sensitivity and spatial frequency of visual stimuli. Nevertheless, the peak of the CSF shifts as viewing distance varies. Therefore, the use of spatial frequency properties of the HVS for IQA tasks is discouraged in [14] because precise knowledge of viewing condition is likely to be unavailable in many practical applications. Furthermore, the computational cost of the schemes with channel decomposition is generally high. Besides the CSF, as the well-known features of the HVS, luminance masking and contrast masking are also widely used in perceptual tasks. For example, a pixel-domain model for just noticeable difference (JND) is introduced in [15] by considering masking effects in texture and edge regions, respectively.

Based on the observation that the HVS is highly adapted to exact structural information, structural similarity (SSIM) index is introduced in [16], where the structural comparison is defined as the correlation coefficient between corresponding patches extracted from the reference and distorted images. This index is attractive due to its mathematical simplicity and effectiveness in predicting subjective rating. Subsequently, some IQA schemes are proposed by extending SSIM, such as multi-scale SSIM [17], complex wavelet SSIM [18], content partitioned SSIM [19], and information content weighting SSIM [20]. In [21], natural scene statistics and mutual information are explored to design the criterion of visual information fidelity (VIF), which has been demonstrated to be equivalent to SSIM [22]. Afterwards, in a number of IQA schemes, various local features are extracted within image patches to measure the distortions. In this paper, we call these features as intra-patch features. A scheme based on the singular value decomposition (SVD) is proposed in [23], where singular values are employed as features. The SVD-based scheme is further improved in [24] by using singular vectors instead of singular values. In [25], sparse feature vectors calculated from independent component analysis serve as the visual responses to image patches. Recently, it has been found that, as an intra-patch feature, image gradient is very effective for IQA tasks [26–33]. In [26] and [27], a modified SSIM is performed on gradient magnitude and edge direction histogram, respectively. Under a similar motivation, the geometric directional distortion model proposed in [28] and [29] is also based on image gradient. In [30], gradient magnitude as well as phase congruency is used to form an objective IQA metric, known as the feature similarity (FSIM) index. In [31], average gradient and edge intensity serve as important parameters to evaluate the quality of remote sensing image. Image gradient can also be incorporated with some psychophysical features of the HVS, including the CSF, perception nonlinearity [32], visibility threshold, masking effects [33], etc. Although the development of objective IQA has been advanced by these schemes, image curvature information is ignored. However, psychophysical studies on scene contour suggest that curvature plays a central role in human perception [34]. In addition, the features are generally extracted and compared within corresponding patches of reference and distorted images. In other words, the visual disparity between a center patch and its spatial neighborhoods is not investigated adequately. In this work, the feature that describes this disparity is named as the inter-patch feature.

In this paper, we propose an FR IQA scheme that compares visual similarity in both the inter-patch and intra-patch ways. The main contributions of this paper are threefold. 1) We investigate the impact of inter-patch feature on the perception of image quality. To this end, a feature vector to describe the disparity between a center patch and its spatial neighborhoods is introduced. The effects of luminance masking and contrast masking are synthetically considered in the design of the inter-patch feature. Moreover, we modify the NCC to make the similarity measurement more reasonable in terms of visual quality. 2) To better measure the perceived similarity within the patches, i.e., the intra-patch similarity, we propose to adaptively use image curvature and gradient comparisons according to local image contents. Specifically, we first partition the image domain into two non-overlapped regions. The partition is based on whether the comparison on image curvature is meaningful. Hence, for one region, merely gradient comparison is performed. For the other region, we compare both the gradient and curvature. 3) An integration strategy to obtain an overall score of image quality is further presented. If the image quality is relative low, we prefer to assign a higher weight to the inter-patch similarity. Otherwise, the intra-patch similarity has a higher weight. The analytical solution to the proposed integration is a nonlinear combination of the inter-patch and intra-patch similarity.

## Motivations

In this section, we provide some observations and thoughts on visual perception and cognition. These observations and thoughts enlighten our work described in this paper.

As mentioned in the previous section, it has been verified that image gradient is an effective intra-patch feature for IQA tasks [26–33]. Besides the change in image intensities, the detection of curvature is also a primary goal of visual analysis [35–36]. Image curvature is expected to convey different information from image gradient, since the same gradient does not mean the same curvature, and vice versa. Essentially, image gradient signifies the first-order information of image derivatives whereas curvature represents the second-order derivatives. The researches on vision have shown that curved lines can be discriminated from straight ones within a spacing that is much smaller than the receptive field of ganglion cells and the physical distance of adjacent cones [37]. This phenomenon demonstrates that the HVS is very sensitive to curvature when human beings perceive scene details and detailed shapes [38]. Furthermore, image curvature has been confirmed to play a part in visual recognition. Objects that are sketched by retaining the points with maximum curvature and joining them with straight lines can still be recognized [39].

Moreover, image curvature or second-order derivatives have been successfully adopted in many image processing systems, e.g., the recent work in [40–42]. Image curvature is used in [40] to distinguish edges and ridges for image interpolation. In our previous work [41], first-order and second-order derivatives are unified in the framework of multi-surface fitting for image super-resolution. In [42], curvature is used to accurately locate the eye center in facial images.

Therefore, in this paper, we employ image curvature as a complement of image gradient to measure the intra-patch similarity for the FR IQA task.

In addition to the intra-patch similarity, we further take advantage of the inter-patch similarity, which is the similarity index of inter-patch features. There exists an intriguing clinical phenomenon known as visual agnosia [43], which refers to the impairment in recognition of visually presented objects. There are two types of visual agnosia, i.e., apperceptive agnosia and associative agnosia. Neither of them can be attributed to the defects in vision, intelligence, or memory. The patients that suffer from apperceptive agnosia can well distinguish the local features of the visual information from the retina. However, they are unable to correctly perceive adjacent features, and thus meaningful objects cannot be correctly formed [44]. This phenomenon implies that the inter-patch feature is crucial in visual cognition.

Recent researches on image recognition and pattern analysis also demonstrate the importance of inter-patch feature in visual cognition [45, 46]. Dominant neighborhood structure is utilized for texture classification purpose in [45], where the inter-patch feature is calculated as the vector with Euclidean distances in luminance space. In [46], the feature of adjacent patches is estimated by ridge regression to extract structural information for the problem of face recognition.

Furthermore, human beings perceive scene in a fashion of non-uniform sampling, and the region around central focus point is perceived accurately without ocular movement [47]. The fovea in the inner retinal surface is responsible for the high-accuracy perception and sees the central visual angle of two degrees [48]. To give an example, we suppose a similar viewing condition as that of the LIVE database [49]: viewing distance is 2–2.5 screen height, and images are displayed at a resolution of 1024 × 768 pixels to fill the screen height. The visual angle *V*, size of visual field *S*, and viewing distance *D* are related by [50]

$$V=2\;\arctan\left(\frac{S}{2D}\right).$$ (1)

Accordingly, when the visual attention is fixed at a given point, the field of sharp central vision is a region with diameter of approximate 54–67 pixels (larger viewing distance results in greater diameter). However, the size of image patches, from which local features are exacted, generally ranges from 5×5 to 13×13 pixels. In this work, the patches are of size 9×9. That is, in common viewing conditions, the image region that can be perceived simultaneously and accurately is much larger than patch sizes. Therefore, it is reasonable and necessary to investigate the inter-patch distortions.

Motivated by the above observations, we take the inter-patch similarity into account for IQA tasks. It is worthwhile to notice that the inter-patch similarity is completely different from the multi-scale strategy, which changes the image dimension to simulate varied viewing conditions. Actually, the proposed scheme can be embedded into a multi-scale framework by assigning relative importance to different scales [17], although we mainly discuss single-scale schemes.

## Methods

**Fig 1:**
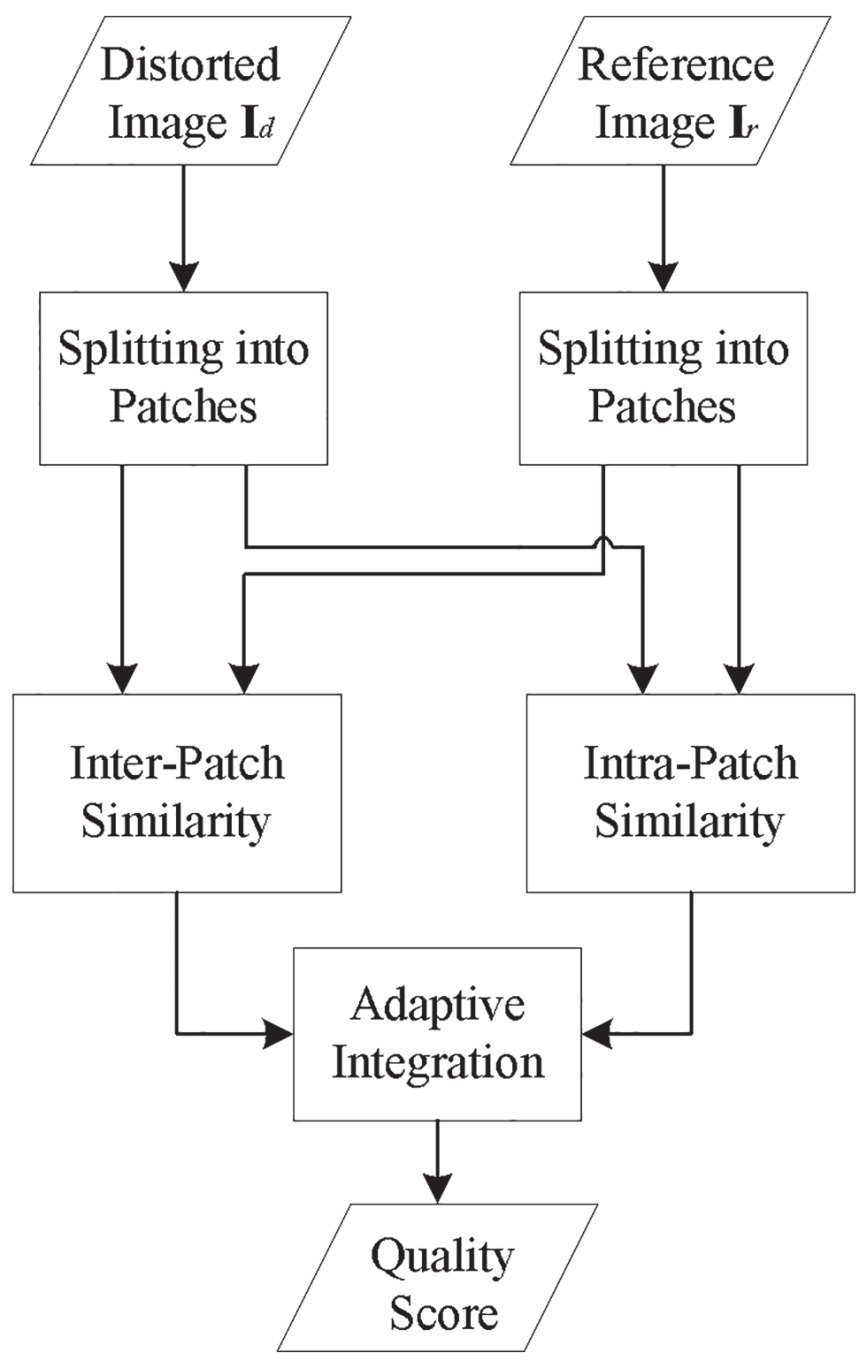
Overall framework of the proposed IQA scheme.

Similar to many existing schemes, we first split images into patches so that a graphical map with distortion measures at each pixel position can be obtained. In the simplified expression, each image patch can be represented by its center pixel. Then, we measure the similarity of the inter-patch and intra-patch features, respectively. Finally, we adaptively integrate the results of the two portions into one single score. The overall framework of the proposed IQA scheme is illustrated in Fig. 1, where some blocks will be introduced in detail in the following.

### 1. Similarity Index of Inter-Patch Feature

To appropriately measure the inter-patch similarity, we first need to describe the inter-patch feature in the reference and distorted images, respectively.

**Fig 2:**
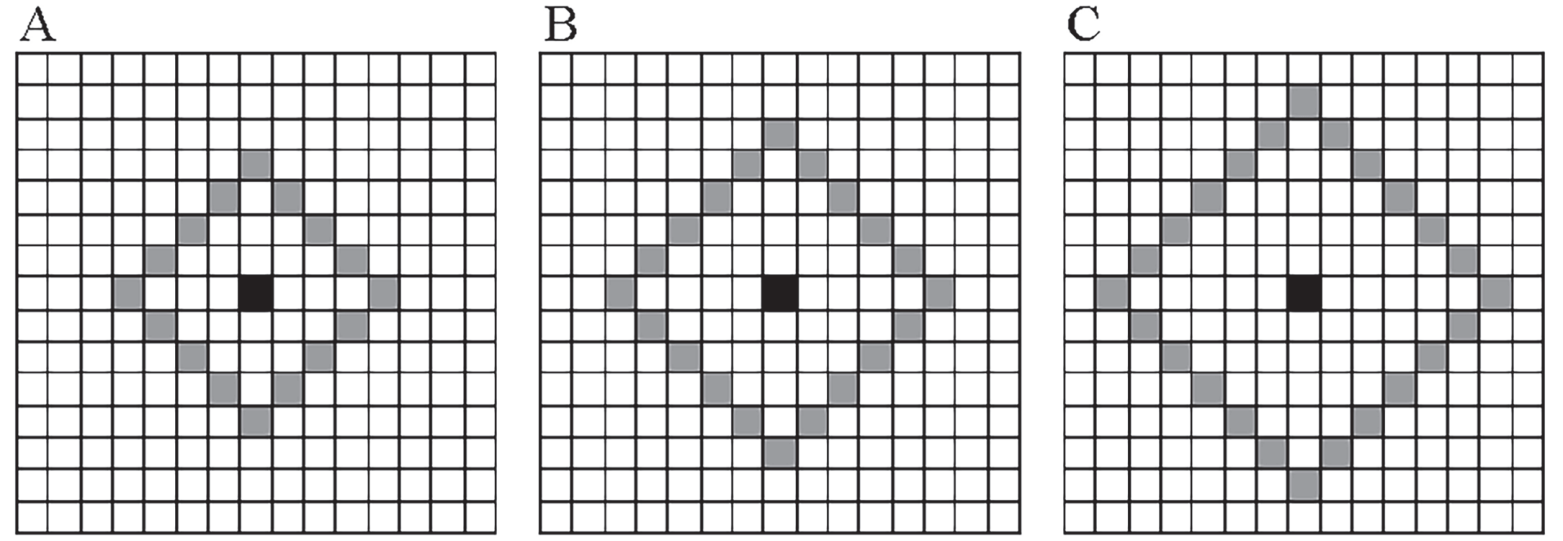
Spatial relationship between a patch and its neighbors for different radius *R*. (A) *R* = 4; (B) *R* = 5; (C) *R* = 6. The center pixel of current patch is indicated by the “black” square, and center pixels of neighbors are denoted by the “grey” squares.

For the patch x_*i*_ (denoted in lexicographic order) centered at the *i*-th pixel *x*_*i*_ in the reference image I_*r*_, its neighbors are the patches centered at adjacent pixels on a diamond of radius *R* using the Manhattan distance. For different radius *R*, the spatial relationship between a patch and its neighbors is illustrated in Fig. 2, where only the center pixels of patches are highlighted. The default value of radius *R* is 6 in this work, and its impact will be presented at the end of results and discussions. It is easy to find that the number of neighbors *N* is proportional to the radius *R*:

N=η⋅R, (2)

where *η* is the proportional coefficient and equals to 4 in the case of Fig. 2. The inter-patch feature is represented by a vector with size of *N*×1, and each element of the vector is the visual disparity between the current patch and its neighbor. For the patch x_*i*_ from the reference image I_*r*_, we denote the feature vector as v_*ri*_. In the perception of visual signal, the luminance masking and contrast masking are two important features of the HVS. The former declares that the disparity in very bright areas is less likely to be visible, and the latter states that the reduction of visibility increases with the strength of the contrast masker [2]. Accordingly, the *j*-th (1≤ *j*≤ *N*) element of the vector v_*ri*_ is calculated as

$$v_{ri}\left(j\right)=sgn\left(\mu_{ri}-\mu_{rij}\right)\cdot \frac{{\left\|x_{i}-x_{ij}\right\|}_{2}^{2}+C_{1}}{M\cdot \max\left(\mu_{ri}^{2},\sigma_{ri}^{2}\right)+C_{1}},$$ (3)

where ||•||_2_ denotes *l*_2_-norm, *sgn*(•) is the signum function, *M* is the number of pixels in each patch, the subscript *r* stands for reference image, *μ*_*ri*_ is the mean intensity of the pixels in the patch x_*i*_, *σ*_*ri*_ is the standard deviation of the pixel intensities in x_*i*_, the patch x_*ij*_ is the *j*-th neighbor of x_*i*_, *μ*_*rij*_ is the mean intensity of the patch x_*ij*_, and the constant *C*_1_ is used to avoid instability when the denominator is very small. In this work, *C*_1_ is set to *M*∙(*K*_1_∙*L*)^2^, where *K*_1_ = 0.01 is a small constant and *L* is the dynamic range of the pixel intensities. For 8-bit grayscale images, *L* equals to 255. By using the signum function *sgn*(•), v_*ri*_(*j*) is positive if the patch x_*i*_ is averagely brighter than its neighbor x_*ij*_. Otherwise, v_*ri*_(*j*) is negative.

For the patch y_*i*_ centered at the *i*-th pixel *y*_*i*_ in the distorted image I_*d*_, we can also represent the inter-patch feature of y_*i*_ as the vector v_*di*_. In the same way as Equation 3, the *k*-th (1≤ *k*≤ *N*) element of v_*di*_ is obtained by

$$v_{di}\left(k\right)=sgn\left(\mu_{di}-\mu_{dik}\right)\cdot \frac{{\left\|y_{i}-y_{ik}\right\|}_{2}^{2}+C_{1}}{M\cdot \max\left(\mu_{di}^{2},\sigma_{di}^{2}\right)+C_{1}},$$ (4)

where the subscript *d* stands for distorted image, *μ*_*di*_ and *σ*_*di*_ are the mean and standard deviation of the pixel intensities in y_*i*_, the patch y_*ik*_ is the *k*-th neighbor of y_*i*_, and *μ*_*dik*_ is the mean intensity of y_*ik*_. The numerator of Equation 3 and Equation 4 is the total disparity of the pixel intensities in term of square error. In the denominator, the square of the mean intensity is adopted to simulate the effects of luminance masking, and the variance is included to reflect the influence of contrast masking. In addition, when the two masking effects simultaneously exist, the stronger one plays the dominant role. Hence, the maximum operator max(•, •) is further employed to choose the dominant masker.

It is straightforward to employ the NCC to measure the similarity of two vectors. For the vectors v_*ri*_ and v_*di*_, we have

$$NCC\left(v_{ri},v_{di}\right)=\langle \frac{v_{ri}}{{\left\|v_{ri}\right\|}_{2}},\frac{v_{di}}{{\left\|v_{di}\right\|}_{2}}\rangle \approx \frac{v_{ri}^{T}\cdot v_{di}+C_{2}}{{\left\|v_{ri}\right\|}_{2}\cdot {\left\|v_{di}\right\|}_{2}+C_{2}},$$ (5)

where <•, •> is the operator of inner product, and *C*_2_ is a small constant, e.g., 10^–3^, for the stability. The range of the NCC is [–1, 1]. To make the quality score range from 0 to 1, we can linearly map the NCC by

$$Mapped\_NCC\left(v_{ri},v_{di}\right)=\frac{1}{2}\left(1+\frac{v_{ri}^{T}\cdot v_{di}+C_{2}}{{\left\|v_{ri}\right\|}_{2}\cdot {\left\|v_{di}\right\|}_{2}+C_{2}}\right).$$ (6)

However, for IQA tasks, a problem exists in the NCC. From Equation 5 or Equation 6, we can find that the correlation coefficient is rather high if either of the vectors is close to the zero vector. This mathematical phenomenon does not match the visual perception of human beings. Unfortunately, the phenomenon would appear frequently, especially when the distorted image is severely blurred. In severely blurred regions, the disparities of adjacent patches are small. As a consequence, each element of v_*di*_ approximates zero, and the correlation coefficient between this v_*di*_ and any v_*ri*_ is high. Obviously, the distortion is underestimated since the perceptual quality of severely blurred images is generally poor. Therefore, the inter-patch similarity index is estimated by modifying Equation 6 as

$$\begin{array}{l} S_{inter}\left(x_{i},y_{i}\right)=\frac{1}{2}\left(1+\frac{v_{ri}^{T}\cdot v_{di}+C_{2}}{\sqrt{\left({\left\|v_{ri}\right\|}_{2}^{2}+C_{2}\right)\cdot \left({\left\|v_{di}\right\|}_{2}^{2}+C_{2}\right)}}\right)\\ =\frac{1}{2}\left(1+\frac{v_{ri}^{T}\cdot v_{di}+C_{2}}{\sqrt{{\left\|v_{ri}\right\|}_{2}^{2}\cdot {\left\|v_{di}\right\|}_{2}^{2}+2C_{2}\left({\left\|v_{ri}\right\|}_{2}^{2}+{\left\|v_{di}\right\|}_{2}^{2}\right)+C_{2}^{2}}}\right) \end{array},$$ (7)

where *S*_*inter*_(x_*i*_, y_*i*_) is the inter-patch similarity for the patch x_*i*_ and y_*i*_. For the modification in Equation 7, we can have the following two observations. Firstly, the inter-patch features are supposed to be similar, i.e., v_*ri*_ ≈ v_*di*_, if the distorted image region has a high quality. In this case, the mapped NCC can well estimate the similarity of v_*ri*_ and v_*di*_. And *S*_*inter*_ and the mapped NCC are almost the same. More specially, the range of *S*_*inter*_ is still between 0 and 1. And the similarity index *S*_*inter*_ reaches 1 if and only if v_*ri*_ equals to v_*di*_. Secondly, when the inter-patch information is severely lost, v_*di*_ can be regarded as the zero vector. The mapped NCC would underestimate the distortion level, i.e., overestimating the similarity. In this case, *S*_*inter*_ is smaller than the mapped NCC, and thus more reasonable.

**Fig 3:**
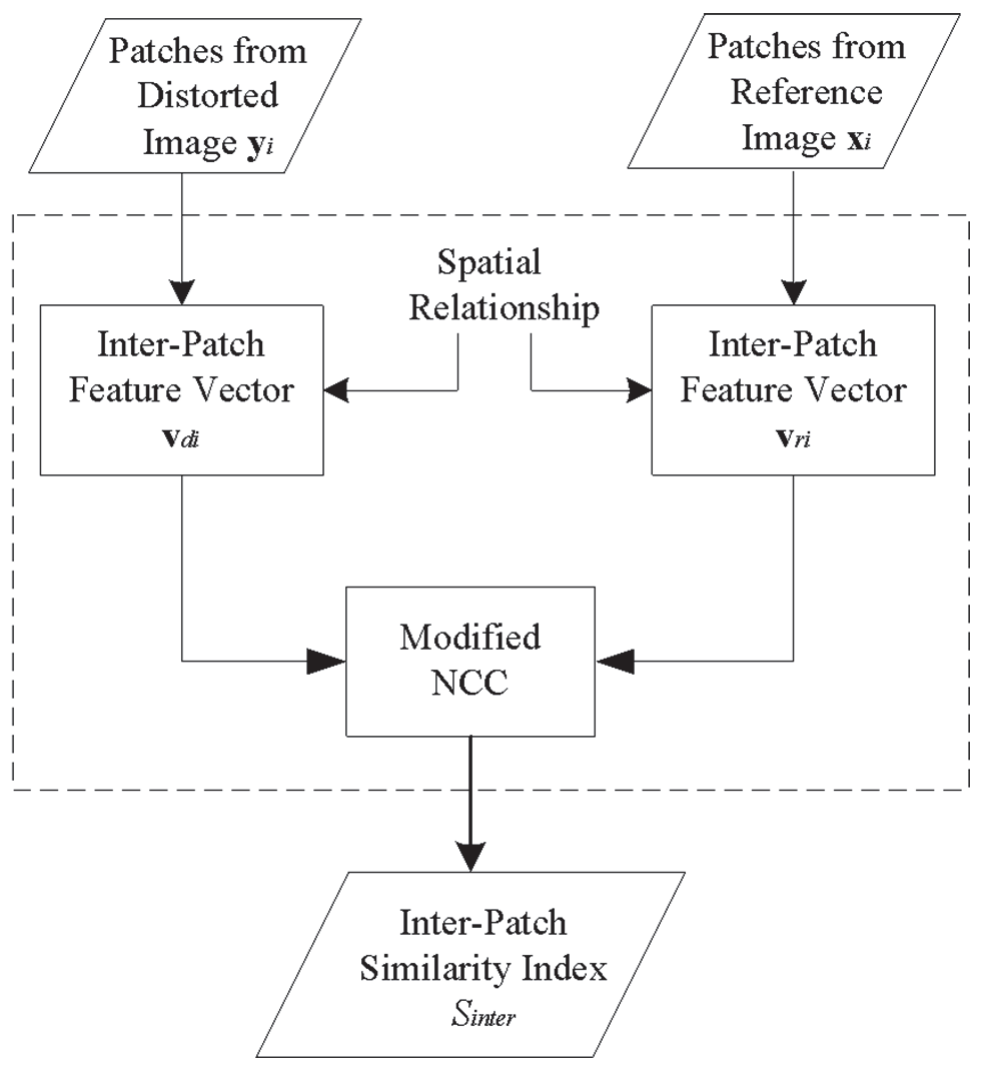
Flowchart of measuring the inter-patch similarity. The dashed block in Fig. 3 replaces block “inter-patch similarity” in Fig. 1.

The flowchart of measuring the inter-patch similarity is illustrated in Fig. 3.

### 2. Similarity Index of Intra-Patch Features

The tests in [51] show that image content has a significant effect on the perceptive image quality. In order to distinguish image patches with different contents, the IQA scheme based on content-partitioned is introduced in [19], where pairs of image patches are classified into different types according to local gradients. Different types of patches are assigned with disparate weights. Essentially, it is merely a strategy for error pooling. To measure the intra-patch similarity, we also partition the images based on the local image contents. Instead of allocating varied weights, we compare different intra-patch features for different types of patches.

In this sub-section, image patches are first classified into two types based on the local gradient and curvature. The image gradient can be estimated by convolving the image with the gradient operator. Various gradient operators are alternative. Among them, we select the Scharr operator, denoted by Φ, as the convolution mask by following the suggestion in [30]. Specifically, we calculate the gradients for the reference image I_*r*_ through

$${Η}_{r}=\Phi *I_{r}=\frac{1}{16}\left[\begin{array}{ccc} 3 & 0 & -3\\ 10 & 0 & -10\\ 3 & 0 & -3 \end{array}\right]*I_{r}\;\text{and}\;V_{r}=\Phi^{T}*I_{r},$$ (8)

where the symbol * means the convolution operation, H_*r*_ and V_*r*_ are the image gradients along horizontal and vertical directions, respectively. And the gradient magnitude G_*r*_ is obtained by

$$G_{r}=\sqrt{H_{r}^{2}+V_{r}^{2}}.$$ (9)

In terms of isophotes, the image curvature K_*r*_ is defined as [40]

$$K_{r}=\left|\frac{-V_{r}^{2}\cdot H_{Hr}+2\cdot V_{r}\cdot H_{r}\cdot V_{Hr}-H_{r}^{2}\cdot V_{Vr}}{{\left(H_{r}^{2}+V_{r}^{2}\right)}^{\frac{3}{2}}}\right|,$$ (10)

where H_H*r*_, V_H*r*_, and V_V*r*_ are the second-order derivatives of I_*r*_. H_H*r*_ and V_H*r*_ are obtained by convolving H_*r*_ with Φ and Φ^*T*^, respectively. Similarly, V_V*r*_ is calculated as Φ^T^*V_*r*_. In Equation 9 and Equation 10, all the operations are performed in the pixel-wise fashion. For the distorted image I_*d*_, we can also calculate the gradient magnitude G_*d*_ and curvature K_*d*_ in the same way as that in Equation 9 and Equation 10.

The main principle of the classification for pairs of image patches is whether the similarity comparison on curvature can be legitimately incorporated. We believe that the following two conditions are necessary for the meaningful comparison on image curvature.

- The gradients from both the reference and distorted images should be visible. The definition of curvature in Equation 10 implies that the estimation of curvature is unstable when the gradient is small. Therefore, the comparison on image curvature is unreliable if either the gradient is invisible. To determine the visibility, we make use of the JND model in [15].
- The curvature from either the reference or distorted images should be smaller than 1. Since the reciprocal of curvature is the radius, the curvature larger than 1 means that the radius is smaller than 1. In the context of digital image, the radius smaller than 1 indicates that we are dealing with a “single dot” isophote. Obviously, the comparison on the curvature of a pair of single dots makes little sense.

**Fig 4:**
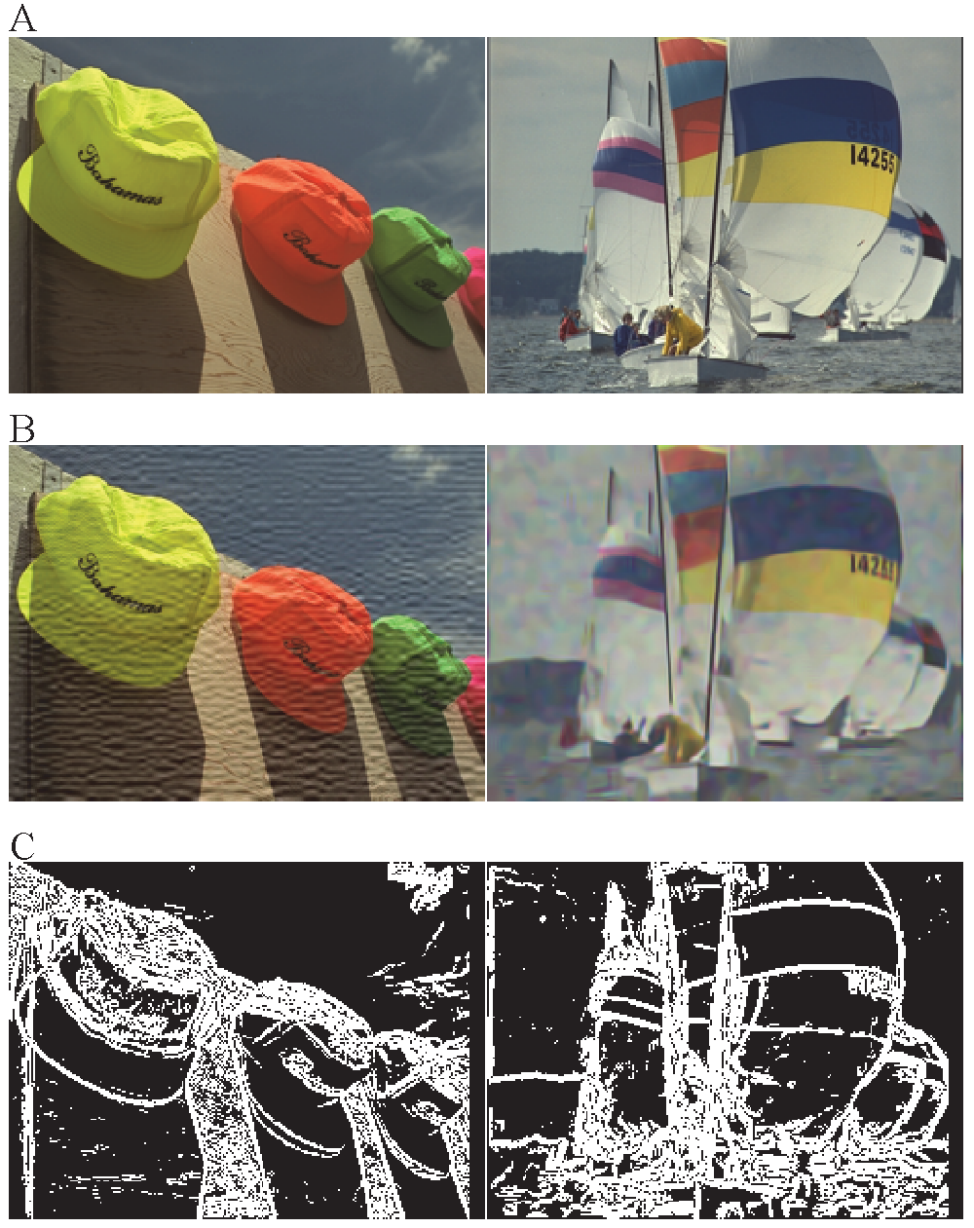
Examples of the image partition, i.e., patch classification. (A) Reference images. (B) Distorted images. (C) Results of our partition. In Fig. 4C, the “white” part is the “Type I” region, and the “black” part is the “Type II” region.

We label the image regions that meet the above conditions as “Type I”, and the remainder regions are labeled as “Type II”. Two examples of the image partition are illustrated in Fig. 4.

For the pair of patches {**x**_*i*_, **y**_*i*_} from the “Type II” region, we merely measure the intra-patch similarity by comparing gradient magnitude:

$$S_{intra}\left(x_{i},y_{i}\right)=\frac{2\cdot G_{r}\left(x_{i}\right)\cdot G_{d}\left(y_{i}\right)+C_{3}}{G_{r}^{2}\left(x_{i}\right)+G_{d}^{2}\left(y_{i}\right)+C_{3}},\;\text{if}\;x_{i}\;\text{and}\;y_{i}\in \;\text{Type}\;\text{II}$$ (11)

where *S*_*intra*_(**x**_*i*_,**y**_*i*_) is the intra-patch similarity for the patch **x**_*i*_ and **y**_*i*_, *x*_*i*_ and *y*_*i*_ represent the center pixels of **x**_*i*_ and **y**_*i*_, and the constant *C*_3_ is used for the stability. In this work, *C*_3_ is set to (*K*_3_∙*L*)^2^, where *K*_3_ = 0.05 is a small constant.

For a pair of patches from the “Type I” region, although at least one of the compared curvatures is smaller than 1, the other one still might be larger than 1. As mentioned above, the curvature larger than 1 means a “single dot” isophote. Since all the “single dot” isophotes in the digital images have similar visual patterns, we should not distinguish the curvatures that are larger than 1. Thus, we modify the isophote curvature by

$$K_{mr}=\min\left(K_{r},1\right)\;\text{and}\;K_{md}=\min\left(K_{d},1\right),$$ (12)

where **K**_*mr*_ and **K**_*md*_ are the adjusted curvatures for the reference and distorted images, respectively. For the pair of patches {**x**_*i*_, **y**_*i*_} from the “Type I” region, we measure the intra-patch similarity using the comparisons on both the gradient magnitude and modified curvature:

$$\begin{array}{r} S_{intra}\left(x_{i},y_{i}\right)={\left(\frac{2\cdot G_{r}\left(x_{i}\right)\cdot G_{d}\left(y_{i}\right)+C_{3}}{G_{r}^{2}\left(x_{i}\right)+G_{d}^{2}\left(y_{i}\right)+C_{3}}\right)}^{\alpha_{1}}\cdot {\left(\frac{2\cdot K_{mr}\left(x_{i}\right)\cdot K_{md}\left(y_{i}\right)+C_{4}}{K_{mr}^{2}\left(x_{i}\right)+K_{md}^{2}\left(y_{i}\right)+C_{4}}\right)}^{\alpha_{2}},\\ \text{if}\;x_{i}\;\text{and}\;y_{i}\;\in \;\text{Type}\;\text{I} \end{array}$$ (13)

where *C*_4_ = 10^–4^ is a constant for the stability to avoid a nearly zero denominator, and *α*_1_ and *α*_2_ are the parameters to adjust the relative importance of gradient and curvature. In this work, we simply set *α*_1_ = *α*_2_ = 0.5 to give them the identical importance.

**Fig 5:**
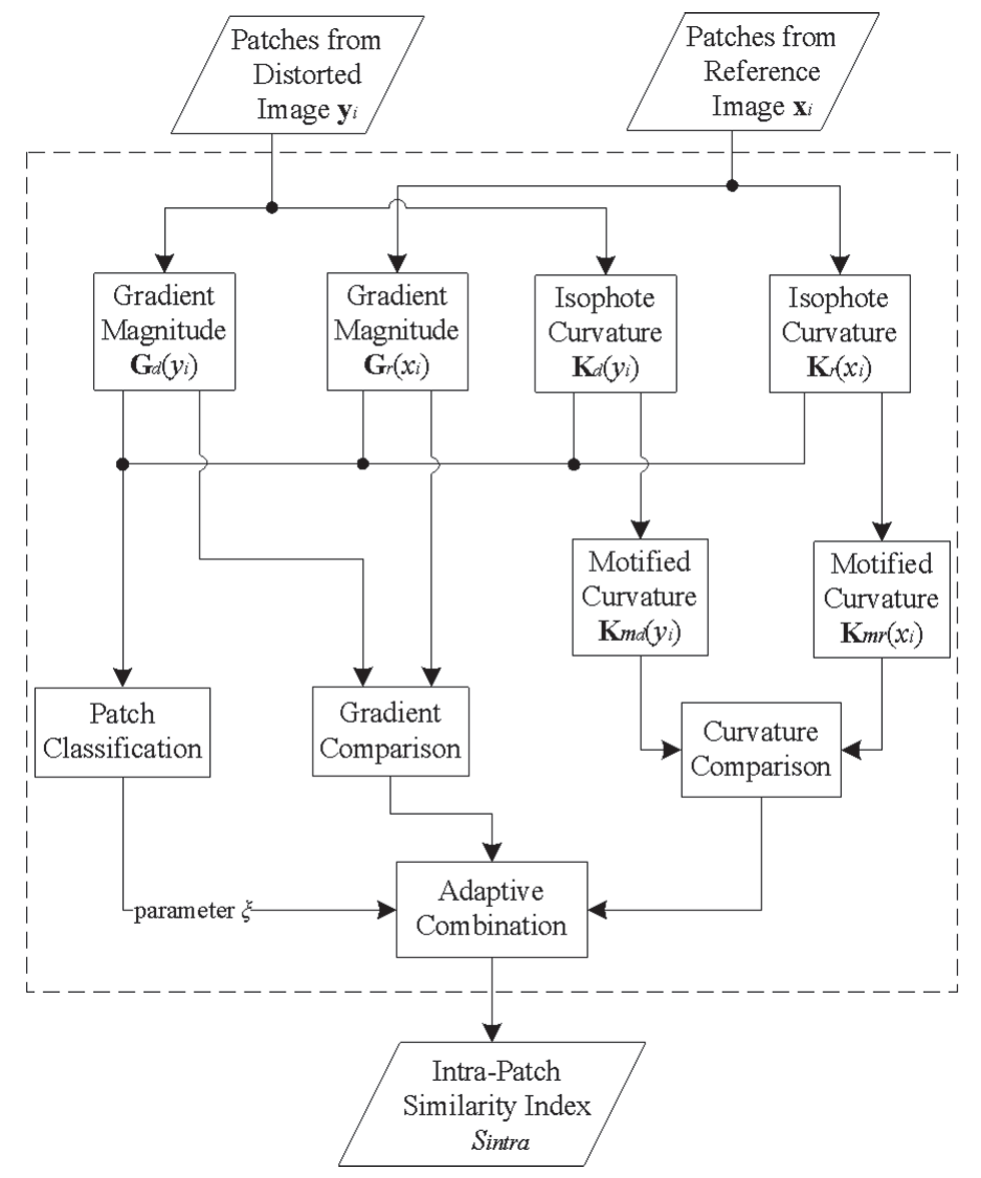
Flowchart of measuring the intra-patch similarity. The dashed block in Fig. 5 replaces block “intra-patch similarity” in Fig. 1.

By observing the forms of Equation 11 and Equation 13, we can readily unify them in the following expression:

$$\begin{array}{r} S_{intra}\left(x_{i},y_{i}\right)={\left(\frac{2\cdot G_{r}\left(x_{i}\right)\cdot G_{d}\left(y_{i}\right)+C_{3}}{G_{r}^{2}\left(x_{i}\right)+G_{d}^{2}\left(y_{i}\right)+C_{3}}\right)}^{\xi }\cdot {\left(\frac{2\cdot K_{mr}\left(x_{i}\right)\cdot K_{md}\left(y_{i}\right)+C_{4}}{K_{mr}^{2}\left(x_{i}\right)+K_{md}^{2}\left(y_{i}\right)+C_{4}}\right)}^{1-\xi }\\ \text{with}\;\xi =\left\{ \begin{array}{rr} 1 & \text{for}\;\text{Type II}\\ 0.5 & \text{for}\;\text{Type I}\; \end{array}\right. \end{array},$$ (14)

where *ξ* is the combination parameter that depends on the patch classification. From Equation 14, we can find that the similarity index of intra-patch features is essentially an adaptive combination of the comparisons on local gradient and curvature. Accordingly, we summarize the procedure of measuring the intra-patch similarity as the flowchart in Fig. 5.

### 3. Overall Objective Score

In the above sub-sections, we have proposed two similarity indices, denoted as *S*_*inter*_ and *S*_*intra*_. The former depicts the inter-patch similarity, and the latter focuses on the intra-patch similarity. We further need to integrate the two portions into an overall measurement.

As mentioned in Motivations, the inter-patch distortions would result in the difficulties in visual cognition. Generally, the images with this kind of distortions are of low qualities. Meanwhile, the intra-patch distortions may be merely caused by the disparity of image details. Therefore, we prefer to assign a relative higher weight to the inter-patch similarity index *S*_*inter*_ if the image quality is relative poor. Conversely, if the image patch is of good quality, the intra-patch similarity index *S*_*intra*_ should have a relative higher weight. Hence, we set the weight of *S*_*intra*_ in the integration is proportional to the integrated index *S*_*I*_. Specifically, for the given patch **x**_*i*_ and **y**_*i*_, the integrated index *S*_*I*_(**x**_*i*_, **y**_*i*_) is related with *S*_*inter*_ and *S*_*intra*_ by:

$$S_{I}=\gamma \cdot S_{I}\cdot S_{intra}+\left(1-\gamma \cdot S_{I}\right)\cdot S_{inter},$$ (15)

where *S*_*I*_, *S*_*inter*_, and *S*_*intra*_ are the abbreviations of *S*_*I*_(**x**_*i*_, **y**_*i*_), *S*_*inter*_(**x**_*i*_, **y**_*i*_), and *S*_*intra*_ (**x**_*i*_, **y**_*i*_), respectively, and *γ* (0<*γ* **≤**1) is the proportional coefficient to relate the weight and *S*_*I*_. In our experiments, the default value of *γ* is 0.8. The impact of this coefficient will be discussed at the end of the next section. Through an algebraic transformation of Equation 15, we have

$$S_{I}=\frac{S_{inter}}{1+\gamma \left(S_{inter}-S_{intra}\right)}.$$ (16)

**Fig 6:**
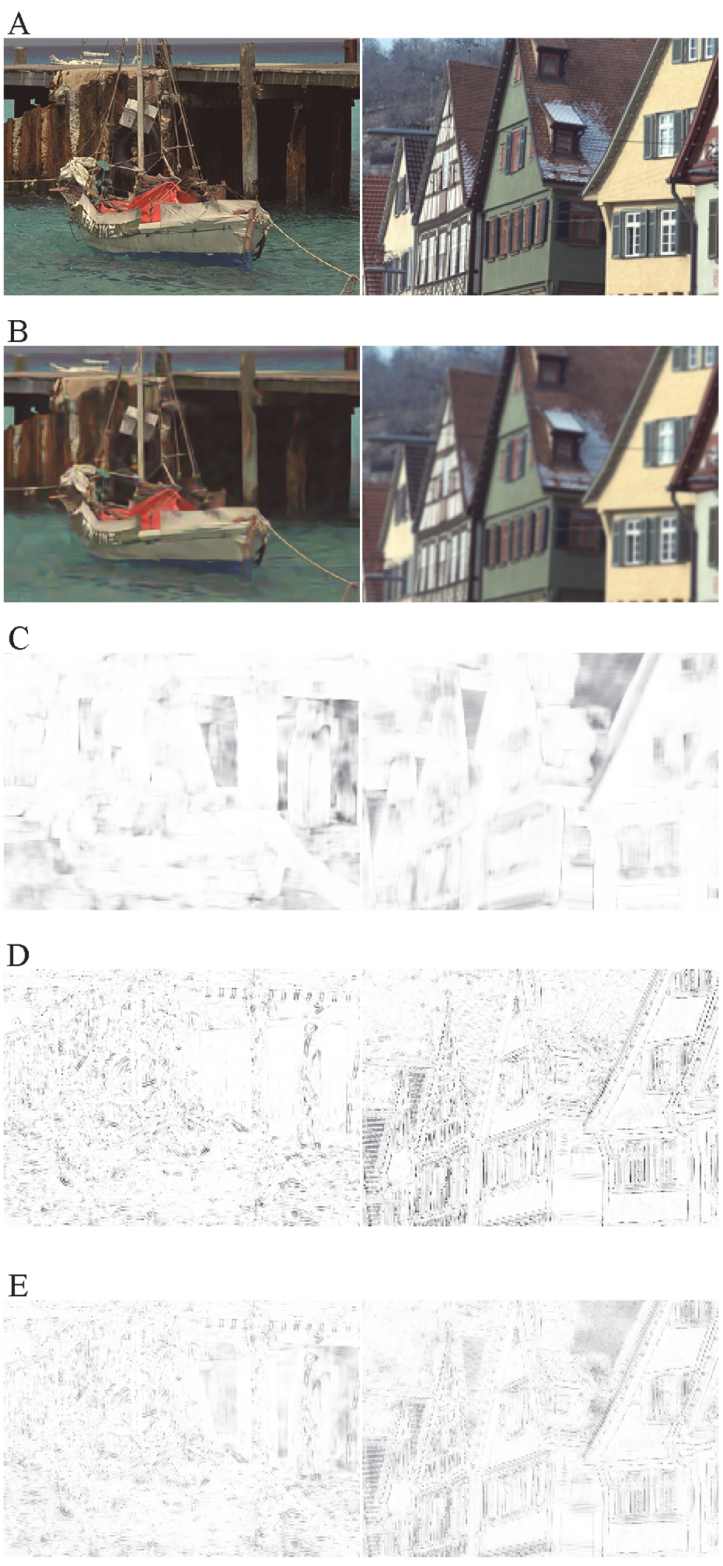
Examples of *S*_*inter*_, *S*_*intra*_, and *S*_I_ maps. (A) Reference images. (B) Distorted images. (C) Map of inter-patch similarity index. (D) Map of intra-patch similarity index. (E) Map of integrated similarity index. For Fig. 6C-6E, the similarity indices are linearly mapped to [**0**, **255**] for visible exhibition.

Under conditions that *S*_*inter*_, *S*_*intra*_ and *γ* range from 0 to 1, it is easy to prove that the range of *S*_*I*_ calculated by Equation 16 is still from 0 to 1. The illustration in Fig. 6 shows two examples of *S*_*inter*_, *S*_*intra*_, and *S*_*I*_ maps. From Fig. 6, we can observe that the responses of *S*_*inter*_ and *S*_*intra*_ have great differences, e.g., *S*_*intra*_ is sensitive to the distortions on the details of sea waves while *S*_*inter*_ is not. There exists the complementarity between the inter-patch similarity and the intra-patch similarity. As was expected, the integrated similarity index *S*_*I*_ is more comprehensive.

To get a single score *q*(**I**_*r*_, **I**_*d*_), we average the integrated index of each pair of patches:

$$q\left(I_{r},I_{d}\right)=\frac{1}{N_{p}}\sum_{i=1}^{i=N_{p}}S_{I}\left(x_{i},y_{i}\right).$$ (17)

where *N*_*p*_ is the number of patch pairs in the reference and distorted images.

## Results and Discussions

In this section, we will provide the experimental results of the proposed scheme. Here our scheme is merely performed on the luminance component of images in a single-scale fashion. For color images, we transform them to the grayscale version before quality assessment. To determine the proper scale, we follow the suggestion of Wang [52], i.e., down-sampling the original images by a fact of *E*:

$$E=\max\left(1,\mathrm{round}\left(N_{h}/256\right)\right),$$ (18)

where round(•) is the function to return the nearest integer of its argument, and *N*_*h*_ is the number of pixels in image height or width.

### 1. Databases and Performance Measures

Our experiments are mainly conducted on six subject-rated and publicly available databases, i.e., TID2008 [53], CSIQ [54], LIVE [49], IVC [55], MICT [56], and A57 [57]. Some important information about these databases, including the numbers of reference and distorted images, is listed in Table 1. In these databases, subjective ratings of all the distorted images are provided to serve as the ground truth in the performance comparison. And the subjective scores are given in the form of either mean opinion score (MOS) or differential mean opinion score (DMOS). To analyse the performance in a common space and remove the nonlinearity caused in the process of subjective rating, we need to apply a regression analysis to non-linearly relate the objective scores, i.e., IQA scheme outputs, and the subjective scores. Following the Video Quality Experts Group tests and validation method [58], we utilize the five-parameter logistic function as:

$$p\left(q\right)=\beta_{1}\cdot \left(\frac{1}{2}-\frac{1}{1-\exp\left(\beta_{2}\cdot \left(q-\beta_{3}\right)\right)}\right)+\beta_{4}\cdot q+\beta_{5},$$ (19)

where *β*_1_, *β*_2_, *β*_3_, *β*_4_, and *β*_5_ are the parameters to be fitted by minimizing the MSE between the mapped values *p* and the subjective scores.

**Table 1:** Some information about databases.

Database	Reference images	Distorted images	Distortion types	*F-critical*
TID2008	25	1700	17	1.083
CSIQ	30	866	6	1.118
LIVE	29	779	5	1.125
IVC	10	185	5	1.274
MICT	14	168	2	1.290
A57	3	54	6	1.571

The performance comparisons of the IQA schemes are based on four widely-used criteria, which are known as Spearman rank order correlation coefficient (SROCC), Kendall rank order correlation coefficient (KROCC), Pearson linear correlation coefficient (PLCC), and root mean squared error (RMSE). The first two criteria evaluate prediction monotonicity, and the other two are employed to measure prediction accuracy [58]. Since SROCC and KROCC merely depend on the rank of data, we can calculate them using the IQA scheme outputs *q* and MOS/DMOS. For the calculation of PLCC and RMSE, the nonlinear regression given in Equation 19 is necessary. That is, PLCC and RMSE are obtained by comparing the mapped values *p* with MOS or DMOS. A better IQA scheme is considered to have higher SROCC, KROCC, and PLCC, as well as lower RMSE.

### 2. Overall Performance Comparison

**Fig 7:**
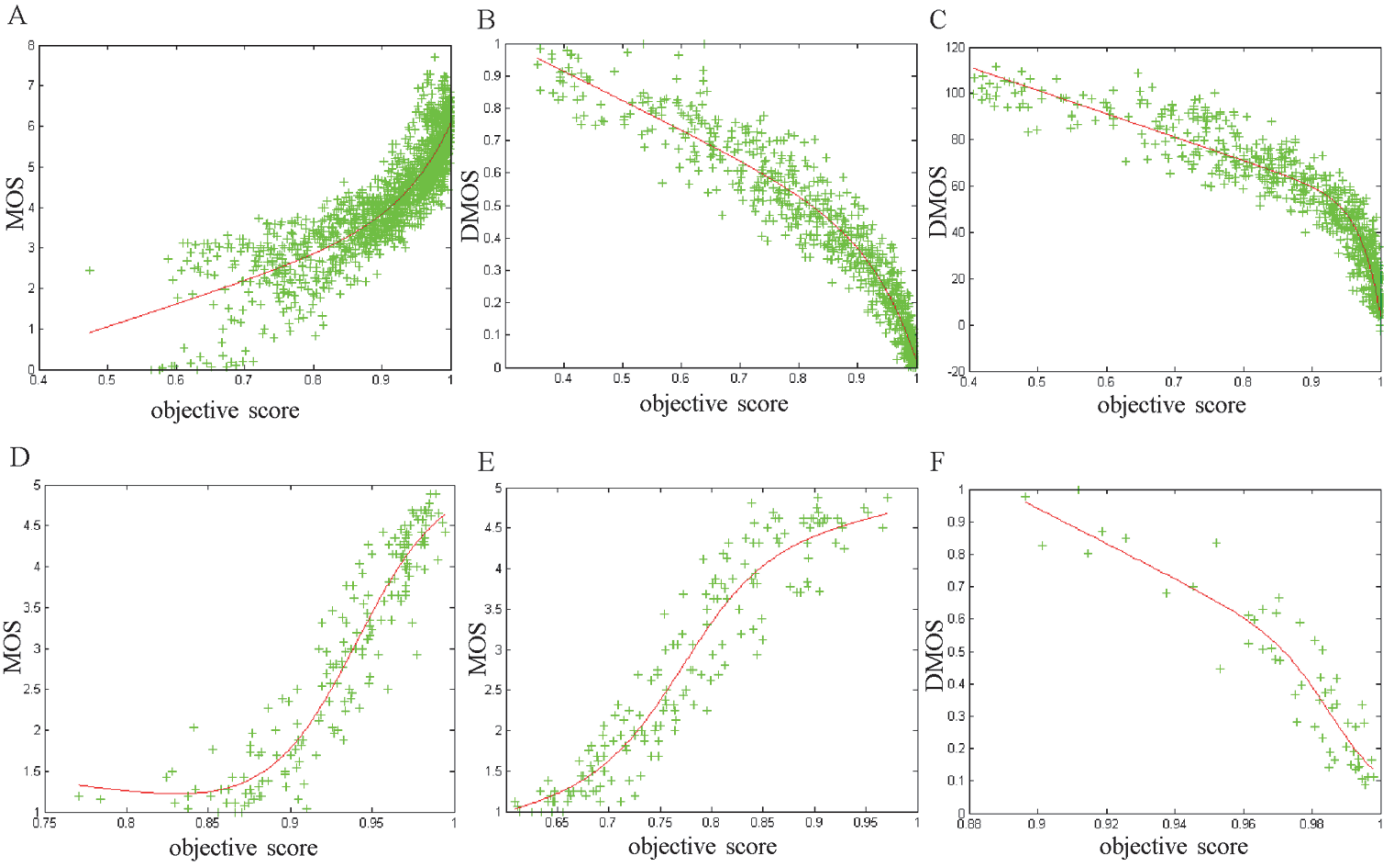
Scatter plots of our IQA scheme on six image databases. (A) TID 2008. (B) CSIQ. (C) LIVE. (D) IVC. (E) MICT. (F) A57.

The scatter plots of the proposed IQA scheme are shown in Fig. 7, where the abscissa denotes the objective scores *q* and the ordinate is the MOS or DMOS. In Fig. 7, each cross-shaped point represents a pair of reference and distorted images.

In Table 2, the proposed scheme is compared with some classical or state-of-the-art IQA schemes, including PSNR, SSIM [16], VIF [21], VSNR [11], FSIM [30], gradient similarity (GSIM) [33], and sparse feature fidelity (SFF) [25]. The two best results across the eight schemes are highlighted in boldface. From Table 2, we can see that the proposed scheme perform consistently well on the six databases. On TID2008 and MICT databases, our scheme has a clear advantage over the compared schemes. On CSIQ and IVC databases, our scheme is still of the best performance, although its superiorities over SFF on CSIQ database and GSIM on IVC database are slight. On LIVE database, the performance of the proposed scheme is comparable with that of FSIM and SSF. On A57 database, VSNR has the best performance. However, its performance is relatively poor on other databases. Moreover, the averaged results on six databases are provided in Table 3, where the best results are highlighted in boldface. The average values are calculated in two cases. In the first case, the results are directly averaged regardless of the database size. In the second case, we assign the databases with different weights that are proportional to the number of distorted images in each database (see Table 1 for the specific numbers). It is worthwhile to notice that the ranges of RMSE values are not the same on the six databases. Consequently, we normalize each RMSE value by dividing it by the RMSE value of PSNR on the same database. Taking the directly averaged RMSE value of SSIM for example, we obtain the normalized RMSE (NRMSE) as

$$\mathrm{NRMSE}\left(\mathrm{SSIM}\right)=\frac{1}{\#(\Omega )}\cdot \sum_{\varphi \in \Omega }\frac{{\mathrm{RMSE}}_{\varphi }(\mathrm{SSIM})}{{\mathrm{RMSE}}_{\varphi }(\mathrm{PSNR})},$$ (20)

where *φ* denotes a certain database, Ω represents the set of databases, and #(Ω) is the number of the elements in the set Ω. In our experiments, Ω is the set {‘TID2008’; ‘CISQ’; ‘LIVE’; ‘IVC’; ‘MICT’; ‘A57’}, and #(Ω) equals 6. As the same with RMSE, lower NRMSE implies the better performance. From the two kinds of average values shown in Table 3, we can conclude that the average performance of the proposed scheme is the best.

**Table 2:** Overall performance comparison for IQA schemes.

Database	Metric	PSNR	SSIM	VIF	VSNR	FSIM	GSIM	SFF	Proposed
TID2008	SROCC	0.5245	0.7749	0.7496	0.7046	**0.8805**	0.8554	0.8767	**0.8952**
	KROCC	0.3696	0.5768	0.5863	0.5340	**0.6946**	0.6651	0.6882	**0.7098**
	PLCC	0.5309	0.7732	0.8090	0.6820	0.8738	0.8462	**0.8817**	**0.8946**
	RMSE	1.1372	0.8511	0.7888	0.9815	0.6525	0.7151	**0.6333**	**0.6085**
CSIQ	SROCC	0.8057	0.8756	0.9193	0.8106	0.9242	0.9126	**0.9627**	**0.9640**
	KROCC	0.6080	0.6907	0.7534	0.6247	0.7567	0.7403	**0.8281**	**0.8283**
	PLCC	0.8001	0.8612	0.9277	0.8002	0.9120	0.8979	**0.9643**	**0.9649**
	RMSE	0.1575	0.1334	0.0980	0.1575	0.1077	0.1156	**0.0695**	**0.0690**
LIVE	SROCC	0.8755	0.9479	0.9631	0.9274	**0.9634**	0.9554	0.9649	**0.9737**
	KROCC	0.6864	0.7963	0.8270	0.7616	**0.8337**	0.8131	**0.8365**	0.8276
	PLCC	0.8721	0.9449	0.9598	0.9231	0.9597	0.9437	**0.9632**	**0.9640**
	RMSE	13.368	8.9455	7.6734	10.506	7.6780	9.0376	**7.3460**	**7.3266**
IVC	SROCC	0.6885	0.9018	0.8966	0.7983	0.9262	**0.9294**	0.9249	**0.9304**
	KROCC	0.5220	0.7223	0.7165	0.6063	0.7564	**0.7626**	0.7553	**0.7682**
	PLCC	0.7199	0.9119	0.9028	0.8032	0.9376	**0.9399**	0.9324	**0.9404**
	RMSE	0.8456	0.4999	0.5239	0.7258	0.4236	**0.4160**	0.4404	**0.4159**
MICT	SROCC	0.6130	0.8794	0.9086	0.8614	0.9059	**0.9233**	0.8992	**0.9388**
	KROCC	0.4447	0.6939	0.7329	0.6762	0.7302	**0.7541**	0.7217	**0.7818**
	PLCC	0.6426	0.8887	0.9144	0.8710	0.9078	**0.9287**	0.9030	**0.9445**
	RMSE	0.9588	0.5738	0.5066	0.6147	0.5248	**0.4640**	0.5378	**0.4113**
A57	SROCC	0.6189	0.8066	0.6223	**0.9355**	0.9181	0.9002	**0.9190**	0.9081
	KROCC	0.4309	0.6058	0.4589	**0.8031**	0.7639	0.7205	**0.7694**	0.7331
	PLCC	0.6587	0.8017	0.6158	**0.9472**	0.9252	0.8976	**0.9449**	0.9274
	RMSE	0.1849	0.1469	0.1936	**0.0781**	0.0933	0.1084	**0.0805**	0.0979

**Table 3:** Average performance on six databases.

Averaging Manner	Metric	PSNR	SSIM	VIF	VSNR	FSIM	GSIM	SFF	Proposed
Direct Average	SROCC	0.6877	0.8644	0.8432	0.8396	0.9197	0.9127	0.9246	**0.9350**
	KROCC	0.5103	0.6810	0.6792	0.6677	0.7559	0.7426	0.7665	**0.7748**
	PLCC	0.7040	0.8636	0.8549	0.8378	0.9194	0.9090	0.9316	**0.9393**
	NRMSE	1.0000	0.7081	0.6808	0.7618	0.5641	0.6002	0.5108	**0.4953**
Weighted Average	SROCC	0.6757	0.8455	0.8456	0.7903	0.9117	0.8967	0.9189	**0.9313**
	KROCC	0.5022	0.6615	0.6860	0.6160	0.7435	0.7228	0.7573	**0.7680**
	PLCC	0.6800	0.8416	0.8743	0.7776	0.9059	0.8874	0.9220	**0.9302**
	NRMSE	1.0000	0.7409	0.6463	0.8621	0.5935	0.6491	0.5253	**0.5084**

To evaluate the statistical significance of the performance difference between the proposed metric and its competitors, we conduct F-test on the prediction residuals between the mapped objective scores *p* and the subjective ratings. Here, the residuals are supposed to be Gaussian. Let *F* denote the ratio between two residual variances, of which the larger one is set as the numerator. The judgment threshold is denoted as *F-critical*, whose value is calculated based on the number of residuals and a given confidence level. The values of *F-critical* with 95% confidence are listed in Table 1 for each database. If *F* is larger than *F-critical*, the difference between the two metrics is believed to be significant at the specified confidence level. In Table 4, we compare the proposed metric with other metrics in the context of statistical significances. In each entry of Table 4, the symbol “1”, “−”, or “0” means that the proposed metric is statistically (with 95% confidence) better, indistinguishable, or worse than the corresponding metric, respectively. Table 4 shows that the proposed scheme statistically outperforms most of compared schemes.

**Table 4:** Performance comparison based on statistical significance.

Database	PSNR	SSIM	VIF	VSNR	FSIM	GSIM	SFF
TID2008	1	1	1	1	1	1	1
CSIQ	1	1	1	1	1	1	−
LIVE	1	1	−	1	−	1	−
IVC	1	1	1	1	−	−	−
MICT	1	1	1	1	1	−	1
A57	1	1	1	0	−	−	−

The symbol “1”, “−”, or “0” means that the proposed scheme statistically (with 95% confidence) better, indistinguishable, or worse than the corresponding scheme.

Besides the average and statistical performance, we further evaluate the efficiency of the proposed scheme. The average consuming time for assessing an image in IVC database on a machine with 1.6 GHz Intel core is recorded in Table 5. All the codes are implemented and executed in Matlab environment. As shown in Table 5, the efficiency of our scheme is moderate.

**Table 5:** Consuming time for IQA schemes (in seconds per image).

	PSNR	SSIM	VIF	VSNR	FSIM	GSIM	SFF	Proposed
Time	0.0337	0.0651	3. 8190	0.6569	0.7402	0.1054	0.1694	0.2571

### 3. Performance on Individual Distortion Types

In this experiment, we investigate the performance of IQA schemes on different types of image distortion. The databases TID2008, CSIQ and LIVE were used for this testing, because they contain the most commonly encountered distortion types. The SROCC values are list in Table 6, where AWGN is the abbreviation of additive white Gaussian noise. Here we choose SROCC due to its suitability for measuring a small number of data points and independence with nonlinear regression [25]. The two best results are shown in boldface. From Table 6, we can find that the proposed scheme performs very well in most distortion types. For 12 out of 17 distortion types in TID 2008, 5 out of 6 types in CSIQ, and 3 out of 5 types in LIVE, our scheme has the best or the second best performance.

**Table 6:** SROCC values of IQA metrics for each distortion type.

Database	Distortion Type	PSNR	SSIM	VIF	VSNR	FSIM	GSIM	SFF	Proposed
TID2008	AWGN	**0.9114**	0.8107	0.8799	0.7728	0.8566	0.8577	0.8729	**0.9105**
	AWGN-color	**0.9068**	0.8029	0.8785	0.7793	0.8527	0.8091	0.8625	**0.9001**
	Spatially correlated noise	**0.9229**	0.8144	0.8703	0.7665	0.8483	0.8907	0.8955	**0.9004**
	Masked noise	**0.8487**	0.7795	**0.8698**	0.7295	0.8021	0.7409	0.8363	0.7726
	High frequency noise	**0.9323**	0.8729	0.9075	0.8811	0.9093	0.8936	0.9118	**0.9166**
	Impulse noise	**0.9177**	0. 6732	**0.8331**	0.6471	0.7452	0.7229	0.7488	0.7578
	Quantization noise	0.8699	0.8513	0.7956	0.8270	0.8564	**0.8752**	0.8456	**0.8802**
	Gaussian blur	0.8682	0.9544	0.9546	0.9330	0.9472	**0.9589**	**0.9626**	0.9583
	Denoising	0.9381	0.9530	0.9189	0.9286	0.9603	**0.9724**	0.9383	**0.9658**
	JPEG compression	0.9011	0.9252	0.9170	0.9174	0.9279	**0.9392**	0.9321	**0.9385**
	JP2K compression	0.8300	0.9625	0.9713	0.9515	**0.9773**	0.9759	0.9763	**0.9834**
	JPEG transmission errors	0.7665	0.8678	0.8582	0.8056	0.8708	**0.8835**	0.8572	**0.8914**
	JP2K transmission errors	0.7765	0.8577	0.8510	0.7909	0.8544	**0.8925**	0.8378	**0.8848**
	Non eccentricity noise	0.5931	0.7107	**0.7608**	0.5716	0.7491	0.7372	0.6966	**0.7826**
	Block-wise distortion	0.5852	0.8462	0.8320	0.1926	0.8492	**0.8862**	0.5663	**0.8716**
	Intensity shift	0.6974	**0.7231**	0.5132	0.3715	0.6720	**0.7170**	0.5217	0.6509
	Contrast change	0.6126	0.5246	**0.8190**	0.4239	0.6481	**0.6737**	0.6458	0.6546
CSIQ	AWGN	0.9363	0.8974	0.9571	0.9241	0.9262	0.9440	**0.9672**	**0.9685**
	JPEG compression	0.8882	0.9546	**0.9705**	0.9036	0.9654	0.9632	**0.9786**	0.9675
	JP2K compression	0.9363	0.9606	0.9672	0.9480	0.9685	0.9648	**0.9859**	**0.9764**
	Pink Gaussian noise	0.9338	0.8922	0.9509	0.9084	0.9234	0.9387	**0.9752**	**0.9540**
	Gaussian blur	0.9289	0.9609	**0.9747**	0.9446	0.9729	0.9589	0.9529	**0.9733**
	Global contrast decrements	0.8622	0.7922	0.9361	0.8700	0.9420	**0.9508**	0.9469	**0.9585**
LIVE	JP2K compression	0.9551	0.9614	0.9653	0.9551	**0.9717**	0.9587	0.9641	**0.9809**
	JPEG compression	0.9657	0.9764	0.9793	0.9657	**0.9834**	0.9098	0.9762	**0.9837**
	AWGN	**0.9785**	0.9694	0.9731	0.9785	0.9652	0.9774	0.9549	**0.9783**
	Gaussian blur	0.9413	0.9517	0.9584	0.9413	**0.9708**	0.9517	**0.9751**	0.9589
	Fast fading	0.9027	**0.9556**	0.9321	0.9027	0.9499	0.9399	**0.9536**	0.9517

### 4. Performance of Each Component

In this sub-section, we will discuss about the inter-patch similarity index *S*_*inter*_ and the intra-patch similarity index *S*_*intra*_. For simplicity, we only conducted the testing on TID2008, which is the largest database. The results of each component are given in Table 7, where *S*_*intra*_ (*ξ* ≡ 1) represents that the parameter *ξ* in Equation 14 is consistently set to 1 for both Type I and Type II. By consistently setting *ξ* to 1, we merely perform gradient comparison for the intra-patch similarity index. Moreover, the performance of SSIM, VSNR, VIF, and GSIM is also listed in Table 7. Based on the results in Table 7, we can have the following observations:

**Table 7:** Performance of each component on TID 2008.

Metric	*S*_*inter*_	SSIM	VIF	VSNR	*S*_*intra*_	*S*_*intra*_ (*ξ* ≡ 1)	GSIM	*S*_*I*_
SROCC	0.8331	0.7749	0.7496	0.7046	0.8759	0.8461	0.8554	0.8952
KROCC	0.6326	0.5768	0.5863	0.5340	0.6838	0.6556	0.6651	0.7098
PLCC	0.8157	0.7732	0.8090	0.6820	0.8517	0.8318	0.8462	0.8946
RMSE	0.7762	0.8511	0.7888	0.9815	0.7033	0.7452	0.7151	0.6085

First, the performance of the integrated index *S*_*I*_ is superior to that of *S*_*inter*_ and *S*_*intra*_. This indicates the effectiveness of the integration strategy defined as Equation 16 and the complementarity between *S*_*inter*_ and *S*_*intra*_.

Secondly, the inter-patch similarity index *S*_*inter*_ can achieve promising results without considering any local features within patches. Specifically, *S*_*inter*_ outperforms VSNR, VIF, and SSIM, which are widely-accepted IQA schemes. This demonstrates that the inter-patch information is beneficial to the prediction of image quality.

Thirdly, *S*_*intra*_ achieves better results in comparison with *S*_*intra*_ ( *ξ* ≡ 1) and GSIM, which are merely based on image gradient. Therefore, the incorporation of image curvature is necessary and beneficial.

### 5. Impact of Parameter Values

The impacts of parameters on the performance of our scheme are checked in this sub-section. We discuss two important parameters, i.e., *R* and *γ*, where *R* is the radius to determine the neighbors (mentioned before Equation 2) and *γ* is the proportional coefficient in the integration of *S*_*inter*_ and *S*_*intra*_ (described in Equation 16). This experiment was conducted on six databases, and the SROCC results of weighted average were recorded.

**Fig 8:**
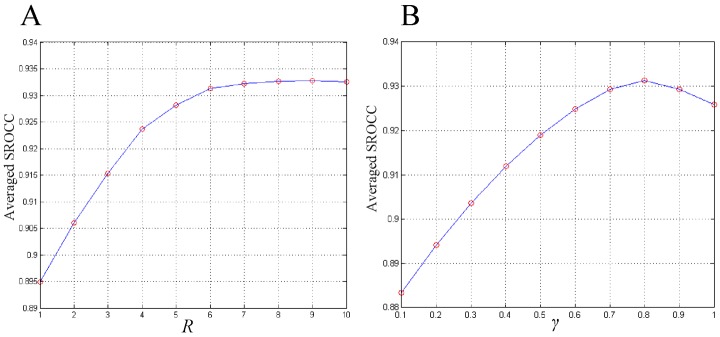
Plots of averaged SROCC values versus parameter values. (A) Impact of parameter *R*. (B) Impact of parameter *γ*.

Fig. 8A plots the averaged SROCC values as a function of *R*, which ranges from 1 to 10 with a step of 1. The value of *γ* is fixed as 0.8 when *R* varies. It is expected that the performance for small *R* is relatively poor, because the inter-patch feature degrades into the feature within patches when the value of *R* is small. As can be observed, performance improves with the increase in *R*. However, the performance improvement is not significant when *R* is larger than 6. Furthermore, there is a tiny decline in the performance when *R* reaches 10. In addition, according to Equation 2, the increase in the number of neighbors is four times faster than that in *R*. It means that the memory space and consuming time increases fast as we enlarge the radius. Hence, it is reasonable to set the default value of *R* to 6.

Fig. 8B plots the averaged SROCC values as a function of *γ*, which ranges from 0.1 to 1 with a step of 0.1. The value of *R* is fixed as 6 when *γ* varies. Based on Fig. 8B, we set the default value of *γ* to 0.8. It is worthwhile to note that larger *γ* does not always mean that the weight of *S*_*intra*_ is larger than that of *S*_*inter*_, since the weight is the product of *γ* and *S*_*I*_. Specially, when the image region is of poor quality, *S*_*inter*_ still has great effects on *S*_*I*_ even if *γ* equals to 1.

## Conclusions

In this paper, an FR IQA scheme based on the inter-patch and intra-patch similarity is introduced. The two similarity indices complement each other. One component aims at measuring the similarity of inter-patch feature, which describes the disparity between a center patch and its spatial neighborhoods. The similarity is measured by the modified NCC to avoid the underestimation of distortions. The other component focuses on measuring the similarity of intra-patch feature. Image patches are classified based on whether the comparison on curvature is reasonable. For one type of patch pairs, we measure the intra-patch similarity on both the curvature and gradient comparison. For another type, merely gradient similarity is included. Moreover, an integration strategy is introduced to get the overall score. A relatively higher weight is assigned to the first component if the image quality is low. Extensive experiments on six publicly available databases show that the proposed scheme is more consistent with subjective evaluations than the compared schemes.

Nevertheless, in this paper, we only measure the similarity on grayscale versions of images. Therefore, the way to take advantage of color information in the proposed scheme needs to be further investigated.
